# Supplementary figures and images for: Regularizing priors for Bayesian VAR applications to large ecological datasets
Source: PeerJ. 2022 Nov 8;10:e14332. doi: 10.7717/peerj.14332 (PMC9651052; doi:10.7717/peerj.14332)

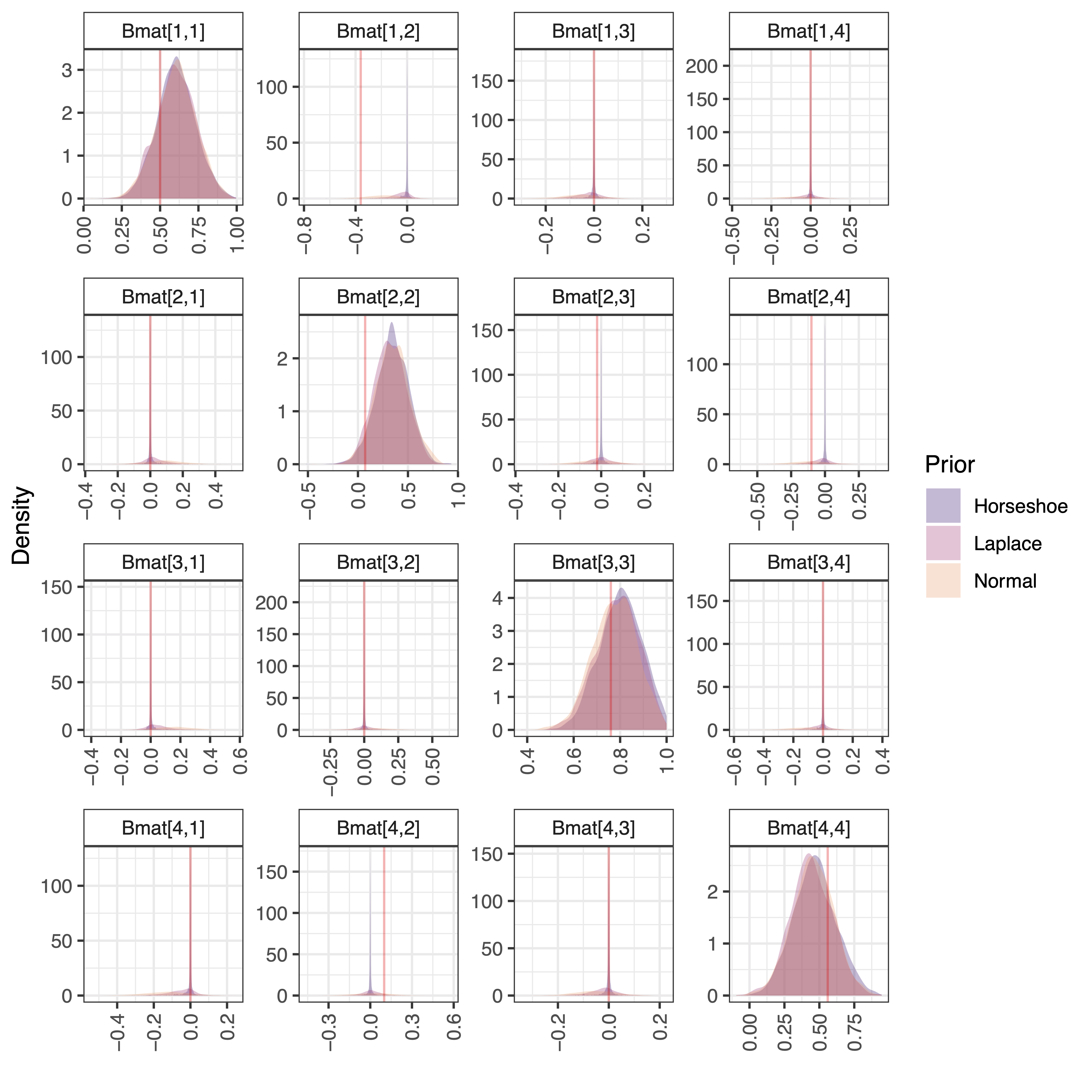

Supplement: Supplemental Information 1 — The true parameter value is shown with a vertical red line in each box. Each element corresponds to the species in Table S1, and the interpretation of elements are the effects of species in each column on the species in each row (e.g., Bmat[1,2] is the effect of small phytoplankton on large phytoplankton). [file peerj-10-14332-s001.png]

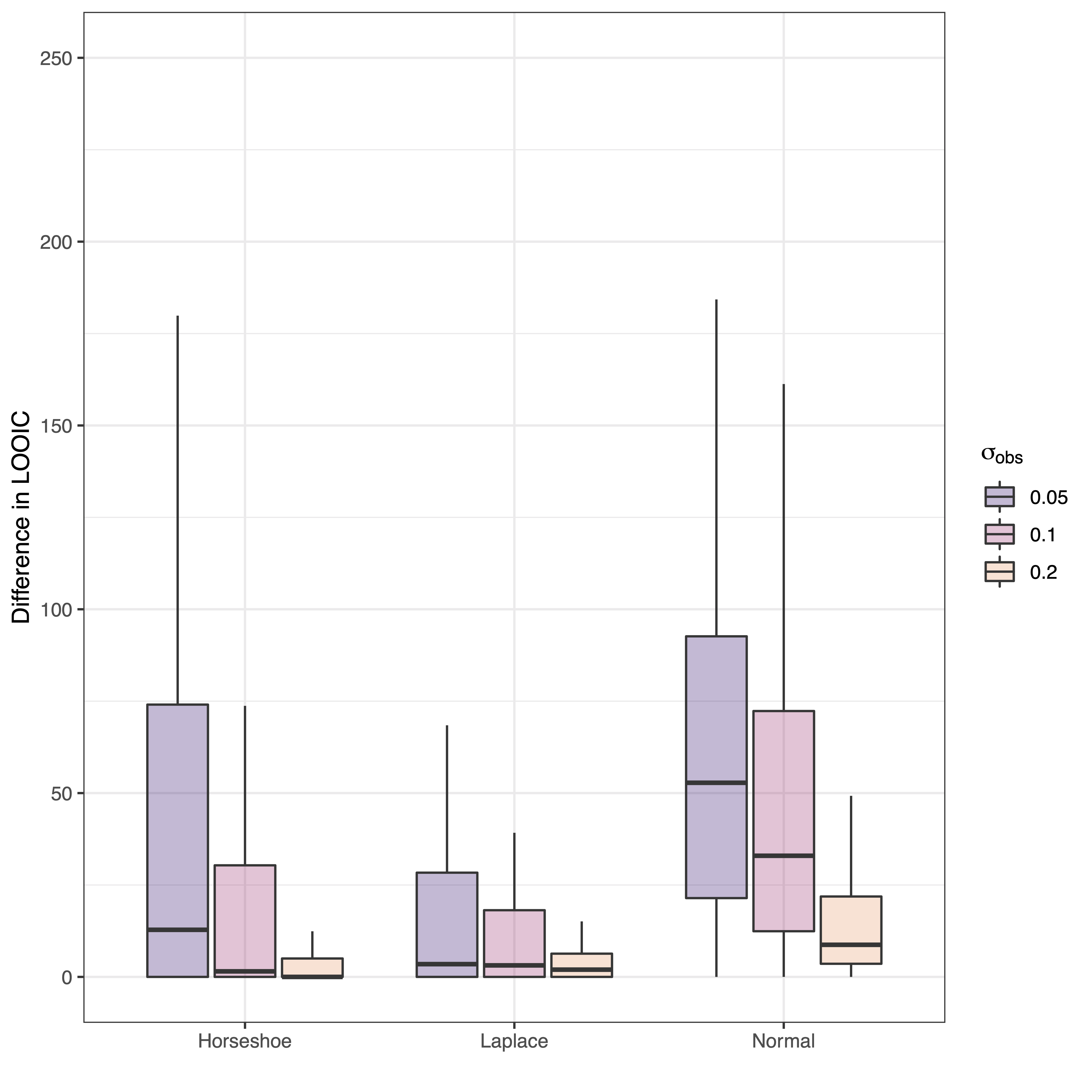

Supplement: Supplemental Information 2 — For each observation error level, 200 random datasets are compared (value of 0 corresponds to the model with highest predictive ability). [file peerj-10-14332-s002.png]
