# Supplementary material for: Regularizing priors for Bayesian VAR applications to large ecological datasets
Source: PeerJ. 2022 Nov 8;10:e14332. doi: 10.7717/peerj.14332 (PMC9651052; doi:10.7717/peerj.14332)
Supplement: Supplemental Information 3 [file peerj-10-14332-s003.docx]

Supplemental Information

Table S1. Interaction matrix from Ives et al. (2003) used for simulations.

|  | Large phytoplankton | Small phytoplankton | Daphina | Non-daphnia |
| --- | --- | --- | --- | --- |
| Large phytoplankton | 0.50 | -0.36 |  |  |
| Small phytoplankton |  | 0.072 | -0.019 | -0.10 |
| Daphina |  |  | 0.76 |  |
| Non-daphnia |  | 0.10 |  | 0.56 |
